# Personalized Three-Dimensional Printing and Echoguided Procedure Facilitate Single Device Closure for Multiple Atrial Septal Defects

**DOI:** 10.1155/2020/1751025

**Published:** 2020-04-25

**Authors:** Ping Li, Fang Fang, Xu Qiu, Nan Xu, Yan Wang, Wen-Bin Ouyang, Feng-Wen Zhang, Hai-Bo Hu, Xiang-Bin Pan

**Affiliations:** ^1^State Key Laboratory of Cardiovascular Disease, Fuwai Hospital, National Center for Cardiovascular Diseases, Chinese Academy of Medical Sciences & Peking Union Medical College, Beijing 100037, China; ^2^Fuwai Yunnan Cardiovascular Hospital, Kunming, Yunnan 650000, China

## Abstract

**Background:**

To evaluate the feasibility of using a single device to close multiple atrial septal defects (ASDs) under the guidance of transthoracic echocardiography (TTE) and with the aid of three-dimensional (3D) printing models.

**Methods:**

Sixty-two patients with multiple ASDs were retrospectively analyzed. Thirty of these patients underwent TTE-guided closure (3D printing and TTE group) after a simulation of occlusion in 3D printing models. The remaining 32 patients underwent ASD closure under fluoroscopic guidance (conventional group). Closure status was assessed immediately and at 6 months after device closure.

**Results:**

Successful transcatheter closure with a single device was achieved in 26 patients in the 3D printing and TTE group and 27 patients in the conventional group. Gender, age [18.8 ± 15.9 (3–51) years in the 3D printing and TTE group; 14.0 ± 11.6 (3–50) years in the conventional group], mean maximum distance between defects, prevalence of 3 atrial defects and large defect distance (defined as distance ≥7 mm), and occluder size used were similarly distributed between groups. However, the 3D printing and TTE group had lower frequency of occluder replacement (3.8% vs 59.3%, *p* < 0.0001), prevalence of mild residual shunts (defined as <5 mm) immediately (19.2% vs 44.4%, *p* < 0.05) and at 6 months (7.7% vs 29.6%, *p* < 0.05) after the procedure, and cost (32960.8 ± 2018.7 CNY vs 41019.9 ± 13758.2 CNY, *p* < 0.01).

**Conclusion:**

The combination of the 3D printing technology and ultrasound-guided interventional procedure provides a reliable new therapeutic approach for multiple ASDs, especially for challenging cases with large defect distance.

## 1. Introduction

Percutaneous transcatheter closure of secundum atrial septal defect (ASD), an established alternative to surgical repair [1], remains challenging in the approximately 10% of cases with multiple ASDs [2]. Use of multiple devices either simultaneously or in staged device closure [3–8] is limited mainly by interference between devices and repeat intervention. On the other hand, use of single device closure for multiple ASDs after balloon atrial septostomy to tear the rim of tissue between the defects or using an oversized device to cover all defects [9, 10] is associated with risk of tearing the atrial septum. Therefore, single device closure that preserves anatomical structure remains the optimal strategy for patients with multiple ASDs. However, this strategy is technically challenging because of inability to determine the target defect for catheter passage and occluder selection, warranting careful interventional planning with comprehensive anatomical information for successful device closure.

Efficiency of single device closure may be increased by use of 3D printed models to inform interventional management, including the selection of optimal target defect and device size, and by using echocardiography for procedure guidance. To this end, use of 3D printing technology [11–15] enables to establish an anatomical and tangible model with the patient's imaging dataset and to visualize directly the patient's true 3D anatomy before the actual procedure [16], while use of echocardiographic guidance may render crossing the target defect less difficult [17–19] than with fluoroscopic guidance alone.

We here report on our preliminary experience with multiple ASDs closure using a single-device strategy with the assistance of 3D printing technology and compare outcomes of 3D printing-based and transthoracic echocardiography (TTE)-guided percutaneous transcatheter closure with those of traditional fluoroscopy-guided closure.

## 2. Methods

### 2.1. Study Patients

This retrospective nonrandomized study enrolled 62 patients with multiple ASDs diagnosed by TTE from April 2016 to November 2017 at our institution. Inclusion criteria were as follows: (1) number of defects ≥2 and (2) largest defect diameter ≥5 mm. Exclusion criteria were as follows: (1) other concomitant congenital cardiac defects requiring catheter or surgical closure and (2) left ventricular ejection fraction <50%.

In 30 out of the 62 patients studied, cardiac computed tomography angiography (CTA) was performed preoperatively to generate personalized heart models with 3D printing. In vitro simulated occlusion then was performed in the 3D printing models for preoperative evaluation. All these patients underwent TTE-guided closure procedure (3D printing and TTE group). The remaining 32 patients with multiple ASDs underwent closure under fluoroscopic guidance (conventional group).

The study protocol was approved by the Institutional Ethics Committee of Fuwai Hospital. Informed consent was obtained from the patients or their legal guardians.

### 2.2. Generation of Heart Model with 3D Printing

**Figure 1:**
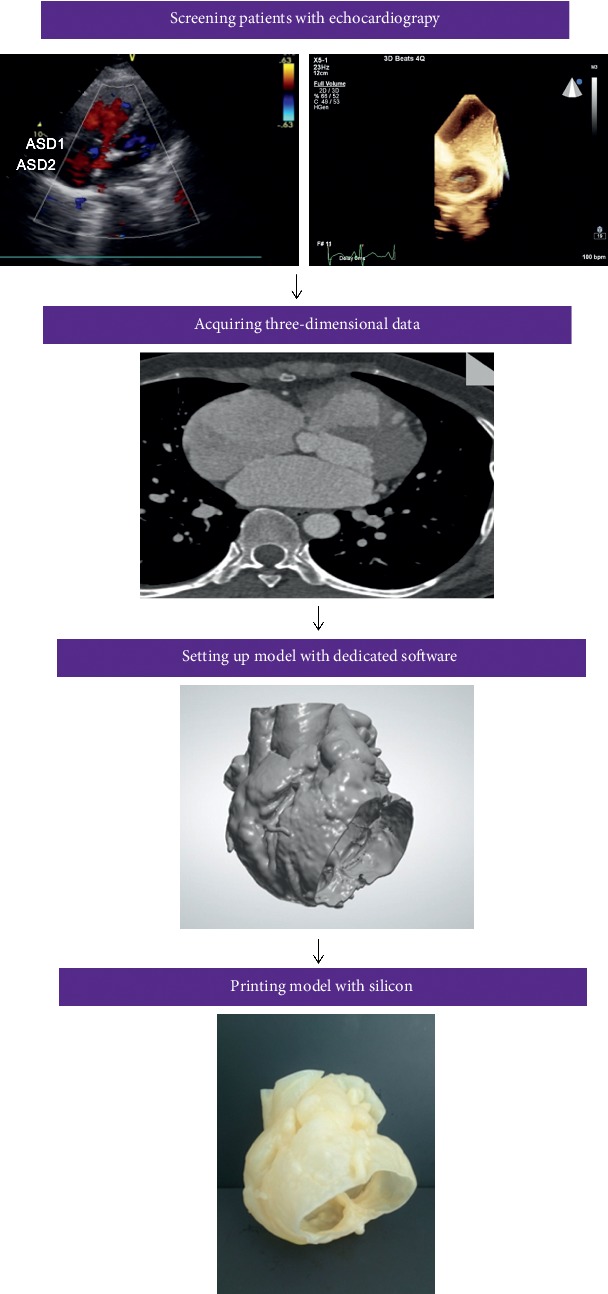
Three-dimensional printing working flow. Simple working flowchart in patients with multiple ASDs, from image acquisition to three-dimensional (3D) printed solid and hollow model.

**Figure 2:**
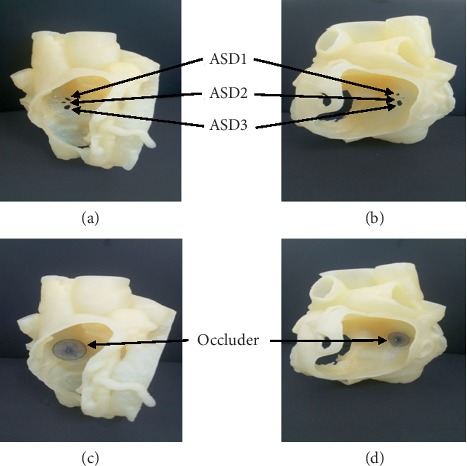
3D printed model of a patient with multiple ASDs. (a) and (b) show the model viewed from left and right atrial sides, respectively. The arrows depict the position of the multiple ASDs. (c) and (d) illustrate the status after occluder deployment in the 3D printing model.

3D printing working flow is displayed in Figure 1. Cardiac CTA scanning was performed in 30 patients. Cardiac CTA images were acquired and reconstructed with 0.625-mm slice thickness at the workstation and then saved in Digital Imaging and Communications in Medicine (DICOM) format. The DICOM file was imported into the Mimics 20.0 software (Materialise NV, Leuven, Belgium). A mask was generated including right/left heart and superior vena cava (SCV)/inferior vena cava (IVC). Then, a cardiac mask was calculated for a 3D model and hollow outside using 3-matic 12.0 software (Materialise NV, Leuven, Belgium) to illustrate the heart structure. The hollow CTA model was verified using the original imaging data. The file was saved in standard tessellation language (.stl format). The final STL files were imported into a 3D printer in a hollow fashion style at a 1 : 1 scale (ProJet MJP 2500 Plus Printer; resolution:800^*∗*^900^*∗*^790DPI, USA) (Figure 2). For simulation of the interventional procedure, soft silicone material was used for printing the personalized heart models. The entire process required a total of approximately 24–48 hours. The cost of the printed model and cardiac CT was 4,200CNY and 995CNY, respectively.

### 2.3. Single-Device Closure Strategy Guided by 3D Printed Heart Model

During preoperative evaluation, occlusion was simulated in vitro using the 3D printing model to inform the closure plan, including the determination of target defect, size selection, and implantation of the device. ASD occluders or patent foramen ovale (PFO) occluders (Lifetech Scientific Co., Ltd. Shenzhen, China) were used in this study. The morphology of the occluder, the coverage of each defect, and the potential disturbance to mitral annulus were observed in the 3D models. Occluder size and type had to meet the following criteria: (1) covering all defects or residual defects ≤1 with residual shunts <5 mm using one occluder with appropriate position and (2) no disturbance between the device and the mitral valve. Based on TTE only for each patient, the diameters of the occluder were estimated empirically by 3 physicians who chose the average size of the occluder.

### 2.4. ASD Closure

**Figure 3:**
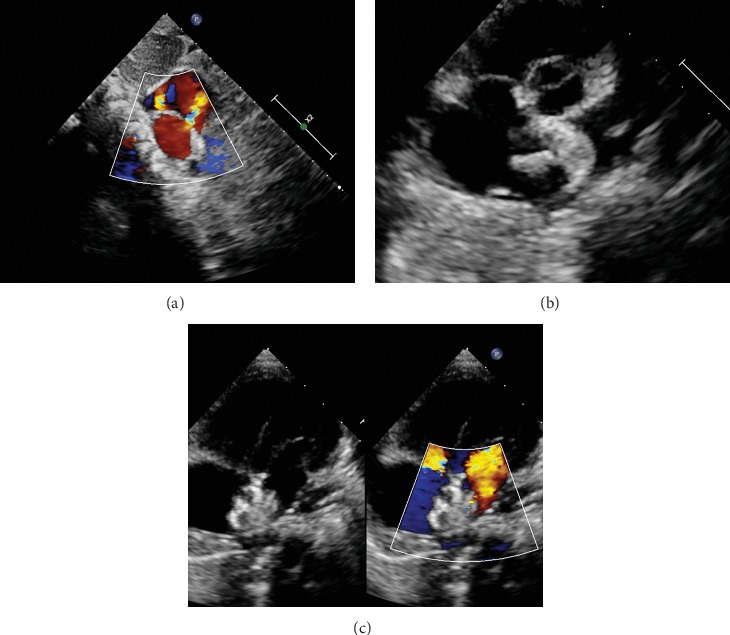
Percutaneous closure of multiple ASDs under TTE guidance. (a) Multiple ASDs image as displayed in subcostal view. (b) The left disc was released (parasternal short-axis view). (c) The ASDs were closed (four-chamber view).

In the 3D printing and TTE group, ASD closure was performed under TTE guidance [19] and local anesthesia. For pediatric patients, sedation titration was utilized with spontaneous breathing. The right femoral vein was punctured, and sheath was inserted. The multipurpose angiographic catheter (MPA2) and the super stiff guide wire (Amplatz Super Stiff™) were inserted via the inferior vena cava to the right atrium, which could be visible on the subcostal view of TTE. The apical four-chamber view and parasternal short-axis view were used for guidance, and the multipurpose catheter was passed through the targeted defect, which was determined using the 3D printing model and intraoperative TTE. The catheter and sheath were withdrawn while maintaining the guide wire in the left atrium. A delivery sheath was inserted into the left atrium along the guide wire. Then, a single septal occluder was inserted for ASD closure under TTE guidance. An ASD occluder or PFO occluder was selected based on the in vitro simulated occlusion in a 3D printing model. After the occluder was successfully implanted, the subcostal, apical four-chamber, and parasternal short-axis views were used to evaluate the device position. Color Doppler assessment also was performed in these views to detect residual shunting, coronary sinus return, and atrioventricular valve function. When the occluder was determined to be implanted in the correct location with a good shape, it was released by rotating the cable counterclockwise under TTE guidance. After device release, reassessment was performed in these echo views (Figure 3).

In the conventional group, multiple ASDs occlusion was performed under fluoroscopic guidance using the single occlusion device. Based on TTE measurements, the single device was selected, equal to or up to 4 mm larger than the main defect [10]. According to experience [10, 20, 21], the device was usually implanted into the largest defect. The occluder was replaced if echography found more than two residual shunts, the residual shunt was >5 mm in diameter, or the device compressed the mitral valve.

All patients received aspirin (3–5 mg/kg for children, 100 mg for adults) orally daily for 6 months after procedure [22]. TTE and electrocardiogram were performed in both groups immediately and at 6 months after procedure. Presence of residual shunt, arrhythmia, or valve dysfunction was noted.

### 2.5. Statistical Analysis

Continuous and categorical variables are expressed as mean ± standard deviation or percentages, respectively. Agreement between device size of 3D printed model and traditional estimation was evaluated by Brand–Altman analysis. Data were analyzed using dedicated software [SPSS Version 17.0, SPSS Inc, Chicago, Illinois, United States; MedCalc software (version 9.2.0.0; Broekstraat, Mariakerke, Belgium)]. *p* value < 0.05 was considered statistically significant.

## 3. Results

### 3.1. Population and Basis Measurements

In the 3D printing and TTE group, of 30 eligible patients, 4 patients (25.8 ± 17.5 years old; 1 female) were excluded because the residual shunt and/or the distance between the occluder and the mitral valve did not meet the criteria during pre-evaluation in the 3D printing heart model before intervention. Surgical closure was performed in these 4 patients, and it was confirmed that they were not good candidates for device closure. The remaining 26 patients [18.8 ± 15.9 years old; 17 (65.4%) females] underwent device closure, and among them, 6 patients had 3 atrial defects (23.1%) (Table 1). According to atrial communication type and form of the defects, PFO occluders were used in 4 patients (15.4%). The average maximum distance between defects was 8.0 ± 3.9 mm, and the prevalence of distance ≥7 mm between the larger defect and the smaller one was 53.8% (14 patients). In the conventional group, 32 patients underwent closure under fluoroscopy guidance. Among them, closure of multiple ASDs failed in 5 patients (28.8 ± 16.3 years old; 1 female). Although we tried 3 different occluders in 1 patient and 4 different occluders in each of the other 4 patients, the closure result was still poor, including two residual shunts or a residual shunt >5 mm, or mitral valve compression by the device. The latter cases were converted to open heart surgery and successfully closed. In the remaining 27 patients [14.0 ± 11.6 years old; female: 15 (55.6%)], successful closure was achieved. Four patients had 3 atrial defects (14.8%); and PFO occluders were used in 3 patients (11.1%). The average maximum distance between the defects was 7.6 ± 3.0 mm, and the prevalence of distance ≥7 mm between the large defect and the smaller one was 40.7% (11 patients). These results were not significantly different from those in the 3D printing and TTE group. Of note, the cost of the 3D printing and TTE group was 32960.8 ± 2018.7CNY, compared with 41019.9 ± 13758.2 CNY in the conventional group, resulting in significant savings (*p* < 0.01).

### 3.2. Comparison of Procedural Results

**Figure 4:**
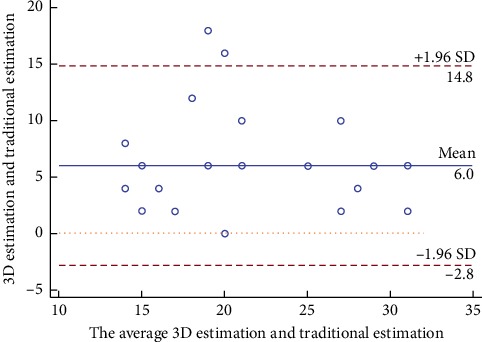
Bland–Altman plot analysis. Bland–Altman plot of empirical estimation versus 3D printed model estimation of occluder size.

In the 3D printing and TTE group, occluder size was consistently similar between 3D printing estimation and actual selection but significantly larger in 3D printing estimation than in conventional empirical estimation (26.0 ± 6.0 mm vs 18.0 ± 6.0 mm, respectively, *p*=0.0007) (Table 1). Mean difference was 6.0 ± 4.5 [95% confidence interval (CI): −3 to 15] by the Brand–Altman analysis (Figure 4). Occluder size in actual selection did not differ significantly between groups (26.0 ± 6.0 mm vs 23.5 ± 5.4 mm, *p*=0.968) (Table 1). One patient (3.8%) in the 3D printing and TTE group underwent occluder replacement once. In contrast, in the conventional method group, 16 patients (59.3%) underwent occluder replacement once in 8 patients (29.6%) and twice and thrice in 4 patients (14.8%) each.

### 3.3. Follow-Up Outcomes

Closure was successful in 26 patients in the 3D printing and TTE group and in 27 patients in the conventional group. In TTE immediately after procedure, prevalence of residual shunts was lower in the 3D printing and TTE group (*n* = 5, 19.2%) than in the conventional group (19.2% vs 44.4%, *p*=0.048); this also was the case at 6-month follow-up: shunting persisted in 2 patients of the 3D printing and TTE group and in 8 patients in the conventional group (7.7% vs 29.6%, *p*=0.042). During procedure or at follow-up in either group, there were no complications, including malposition of devices, aortic/atrial erosion, device thrombosis, atrioventricular block or other arrhythmias, pericardial effusion, or new onset atrioventricular valve dysfunction.

## 4. Discussion

This retrospective study assessed the feasibility of single device closure for multiple ASDs under TTE guidance using patient-specific custom 3D printed models. Furthermore, we compared the new 3D printing and TTE method with conventional fluoroscopy guidance closure. We found that the occulder size measured on the 3D printing model was consistently larger than in the empirical estimation but similar to final clinical selection, indicating more accuracy in portraying multiple ASDs with 3D printing. In addition, residual shunt frequency was lower for the 3D printing and TTE method than the conventional method.

### 4.1. Closure of Multiple Atrial Septal Defects with Single Device

The appropriate therapeutic strategy for multiple ASDs remains controversial. Single device closure remains challenging due to complicated anatomy and technical difficulty. Single device closure was achieved only in those with defect distance <7 mm [10]; however, in the present study, 53 patients successfully underwent transcatheter closure therapy using a single occluder, and the mean maximum defect distance in the two groups was >7 mm. Follow-up data showed that residual shunt volume was significantly reduced or even disappeared at 6 months after procedure, although echocardiography immediately after procedure showed a mild residual shunt. No occluder malposition, atrioventricular block, new onset atrioventricular valve dysfunction, or pericardial effusion occurred during follow-up. These results suggest that interventional therapy with a single occluder for multiple ASDs is feasible, even in patients with a large defect distance.

### 4.2. 3D Printing and TTE Guidance in Multiple Atrial Septal Defects

Successful device closure of atrial communications in multiple ASDs is largely dependent on accurate anatomical assessment [23, 24]. 3D printing model allows to test multiple occluders in the replicated model of the patient's heart before occluder deployment in vivo. In the present study, 5 patients in the conventional group finally failed closure after 3 or 4 occluder replacements; 59.3% of the remaining patients who successfully completed closure also experienced occluder replacement. Four patients in the 3D printing and TTE group were excluded from receiving interventional therapy after the pretest in the 3D printed heart model. Occluders' sizes preestimated by the 3D printed model were similar to the size actually used for patients and larger than the size from conventional empirical estimation. These results indicate that preevaluation using the 3D printed model can avoid unnecessary interventions, the possibility of enlarging ASD by changing occluders and the financial waste of replacing occluders. Therefore, the 3D printed model was extremely helpful in informing interventional management, specifically in determining the most optimal target defect, and the appropriate occluder type and size for multiple ASDs.

Even with a perfect interventional plan with the help of 3D printed models, it remains difficult to improve clinical results due to the inability of distinguishing the position of the target defect using fluoroscopy alone. Consequently, 3D printing technology itself is not likely to change the treatment mode and strategy. Fortunately, percutaneous closure without fluoroscopy use has been making great progress. Echocardiography can be used as the sole imaging tool to guide ASD, VSD, and PDA closure [18, 19, 22]. In this study, we performed percutaneous ASD closure under TTE guidance on the basis of the 3D printed model. Compared with the conventional group, the 3D printing and TTE group showed lower frequency of occluder replacement, lower cost, and lower prevalence of residual mild shunts immediately and at 6 months after procedure. It is important to combine 3D printing with another technique to improve clinical results. 3D printing changed traditional treatments in orthopedics and stomatology [25, 26]; however, it is still not used too often in cardiovascular disease. Unlike teeth and bone, the heart beats every second. In this study, the new treatment strategy for multiple ASDs of combining TTE guidance and 3D printing technology provided more favorable therapeutic efficacy relative to the traditional approach.

### 4.3. Study Limitations

The current study has several limitations including its small sample size and short follow-up duration. Larger studies with longer follow-up period are needed to further investigate the safety of the single device approach for multiple ASDs regardless of distance between fenestrated ASDs. Secondly, obtention of CT datasets for the generation of 3D printed models increases medical risk and cost. Prospective randomized multicenter studies are needed to further validate 3D printing technology based on 3D echocardiographic data.

### 4.4. Impact on Daily Practice

The 3D printed model can help to screen patients who are not suitable for closure. In treatment of multiple ASDs, the new strategy of combining 3D printing technology and TTE-guided interventional procedure yielded lower frequency of occluder replacement, cost, and prevalence of residual mild shunts immediately and 6 months after procedure than traditional fluoroscopy-guided intervention.

## 5. Conclusions

Successful closure of multiple ASDs with defect distance ≥7 mm was achieved using a single device approach aided by the 3D printed model, which can help to screen patients who are not suitable for closure and should receive surgical repair directly. The combination of the 3D printing technology and ultrasound-guided interventional procedure provides a new approach for individualized therapeutic strategy of structural heart disease and in particular a reliable therapeutic method for multiple ASDs, especially for challenging cases with large defect distance.

## Figures and Tables

**Table 1:** Patients' data.

	3D printing and TTE group (*n* = 26)	Conventional group (*n* = 27)	*p* value
Female	17 (65.4%)	15 (55.6%)	0.858

Age (years)	18.8 ± 15.9	14.0 ± 11.6	0.217

Three defects	6 (23.1%)	4 (14.8%)	0.452

Maximum distance (mm) defect	8.0 ± 3.9	7.6 ± 3.0	0.673

Large defect distance (defined as ≥ 7 mm)	14 (53.8%)	11 (40.7%)	0.349

PFO occluder	4 (15.4%)	3 (11.1%)	0.646

Occluder replacement	1 (3.8%)	16 (59.3%)	<0.0001
Once	1 (3.8%)	8 (29.6%)	
Twice		4 (14.8%)	
Thrice		4 (14.8%)	

Occluder size, empirical estimation (mm)	18.0 ± 6.0^*∗*^	—	
Occluder size, 3D model estimation (mm)	26.0 ± 6.0	—	

Occluder size for patients (mm)	26.0 ± 6.0	23.5 ± 5.4	0.968

Intraoperative residual shunt	5 (19.2%)	12 (44.4%)	0.048

Residual shunt at 6 months after procedure	2 (7.7%)	8 (29.6%)	0.042

Cost (CNY)	32960.8 ± 2018.7	41019.9 ± 13758.2	0.0047

Values are expressed as means ± SD, *n* (%). ^*∗*^*p*=0.0007 vs occluder size on 3D model.

## Data Availability

The data used to support the findings of this study are included within the article.
